# Predicting return to use for people with substance abuse disorders

**DOI:** 10.1192/j.eurpsy.2025.783

**Published:** 2025-08-26

**Authors:** L. Mehl-Madrona, B. Mainguy, B. Lamboy, J. Lamboy

**Affiliations:** 1 Native Studies, University of Maine, Orono; 2 Psychiatry Residency, Northern Light Acadia; 3 Wabanaki Health and Wellness, Bangor; 4 Research, Coyote Institute, Orono, United States

## Abstract

**Introduction:**

Substance Use can be a lifelong issue. Recovery is not necessarily easy and not necessarily predictable. Treatment can be expensive and is potentially offset by other influences.

**Objectives:**

We wanted to predict the likelihood of returning to use for Indigenous clients being treated for Substance Use Disorders in the United States.

**Methods:**

We interviewed 35 people who had been diagnosed with a Substance Use Disorder and had been treated in a conventional detox program followed by 30 to 120 days of residential treatment. The participants were obtained by word of mouth referrals, posters and flyers announcing the studiekeeeeeeWe gathered their life stories using a modification of the Northwestern University Life Story Interview which we called the Maine Life Story Interview. We correlated elements from the life story with the likelihood of return to use. By virtue of where the authors worked (Clinics providing services to Indigenous people), all the clients were Indigenous, though we did not aim for that at the outset. Logistic regression methods were used to predict return to use.

**Results:**

The combination of an ACE Score of 5 or greater, a history of substance use for 4 or more years, couipled with returning to the environment of the original substance abuse (physical and/or human) was associated with a 100% probability of return to use regardless of substance used and despite treatment received., With an ACE score of less than 5, the probability of return to use dropped to 80%. The length of treatment before returning to community did not matter. Adverse Childhood Events were measured using the ACE score and resiilience was assessed using a qualitative rating that has been shown to be reliable. Logistic regression confirmed the above -- that the most powerful predictors were returning to the community in which the abuse began and returning to the same social network of people who abused. These variables predicted 84% of the variance. Treatment type or duration did not emerge as significant.

**Conclusions:**

For Indigenous clients, returning to the environment of substance abuse and to the people with whom substance have been used is a powerful factor that overrides the effects of treatment. The higher the ACE score, the more likely the return to use. The length of treatment (ranging from 10 to 130 days) did not seem to matter. Based upon this data, one could argue that tretment should take place in the community from which the person came and be oriented toward changing social networks and eliminating environmental cues. If this is not possible, returning to community appears frought with peril, though inevitable for Indigenous people who need to come home again. Resilience was not a predictive factor once one had returned to the social and environmental locale of the original substance use.

**Disclosure of Interest:**

None Declared

